# Determining Cutoff Point of Ensemble Trees Based on Sample Size in Predicting Clinical Dose with DNA Microarray Data

**DOI:** 10.1155/2016/6794916

**Published:** 2016-12-20

**Authors:** Selen Yılmaz Isıkhan, Erdem Karabulut, Celal Reha Alpar

**Affiliations:** ^1^Vocational School of Social Sciences, Hacettepe University, Ankara, Turkey; ^2^Department of Biostatistics, Faculty of Medicine, Hacettepe University, Ankara, Turkey

## Abstract

*Background/Aim*. Evaluating the success of dose prediction based on genetic or clinical data has substantially advanced recently. The aim of this study is to predict various clinical dose values from DNA gene expression datasets using data mining techniques.* Materials and Methods*. Eleven real gene expression datasets containing dose values were included. First, important genes for dose prediction were selected using iterative sure independence screening. Then, the performances of regression trees (RTs), support vector regression (SVR), RT bagging, SVR bagging, and RT boosting were examined.* Results*. The results demonstrated that a regression-based feature selection method substantially reduced the number of irrelevant genes from raw datasets. Overall, the best prediction performance in nine of 11 datasets was achieved using SVR; the second most accurate performance was provided using a gradient-boosting machine (GBM).* Conclusion*. Analysis of various dose values based on microarray gene expression data identified common genes found in our study and the referenced studies. According to our findings, SVR and GBM can be good predictors of dose-gene datasets. Another result of the study was to identify the sample size of *n* = 25 as a cutoff point for RT bagging to outperform a single RT.

## 1. Introduction

Microarray technology can simultaneously measure the expression levels of thousands of genes in a biological sample. In genome experiments, researchers frequently encounter high-dimensional data with a small sample size [1]. Regression and classification applications created according to classical statistical methods work based on assumptions known as probability distribution models. These assumptions are difficult to satisfy for high-throughput datasets. Therefore, when the probability assumption is unknown, the use of distribution-independent methods is required. Classical statistical methods, such as logistic regression and linear regression analysis, have difficulty in explaining thousands of genes of a small number of individuals. Data mining methods on the other hand conclude the analyses correctly almost requiring no assumption. These methods are useful when there are many explanatory variables available, and they can even be used to examine nonlinear data structures and do not need any particular distribution of the response variable. Various data mining applications, which provide successful results for datasets containing a small number of observations in the high-dimensional space especially in biological applications, have been widely used. Support vector machines (SVMs), decision trees, and boosted trees have recently been used on such data types as alternative tools [2–8]. However, most of them have compared bagging and boosting methods for classification algorithms. For example, Martinez and Suarez examined the effects of the sampling ratio on the properties of the bagging ensembles for classification trees [9]. They analyse bagging in 30 datasets using different sampling ratios from 2% to 120%. Their results demonstrated that using smaller training samples could be useful to improve the generalization performance of the ensemble for several datasets. There is no such study showing how a small sample size affects the regression performances of bagging and boosting.

The aim of this study is to evaluate clinical-dose estimation by genome data (with various sample size) using several data mining techniques. For example, in the International Warfarin Pharmacogenetics Consortium report, “Estimation of the Warfarin Dose with Clinical and Pharmacogenetic Data” whose authors Klein, Altman, and Eriksson became committee members in 2009, therapeutic warfarin dose estimation was obtained using genetic factors, such as gene expression values, and some environmental factors as independent variables [10]. Similarly, various quantitative clinical or chemometrical components were predicted in this study using only gene expression sequences. For this purpose, real datasets containing both gene sequences and clinical-dose measurements related to humans, rat types, and yeast species were used. To avoid making assumptions about the type of relation between the dose values and DNA gene expression data, to cope with high-dimensional or nonlinear data structures, data mining methods such as regression trees (RTs), support vector regression (SVR), RT bagging, SVR bagging, and RT boosting were applied and compared in this study [6, 8].

## 2. Materials and Methods

### 2.1. Preparation of Genetic Data and Feature Selection

Eleven real dose-gene expression datasets were used as real datasets to implement the prediction process. For this purpose, we searched the gene sequences in the GEO database and selected datasets containing a numerical measurement such as dose or concentration. DNA expression profiles of humans, rat types, and yeast species were downloaded from the National Center for Biotechnology Information Gene Expression Omnibus (NCBI GEO). These datasets were used in different studies that examine expression profiles of gene sequences at varying dose or concentration levels (e.g., copper concentration for human and glucose concentration for rat) for different living species. Two of these belong to yeast, seven belong to rats, and the remaining two contain human gene sequences. Our main criterion in the selection of these datasets is that they have both a gene sequence and a numerical response value (e.g., dose). Therefore, a common characteristic of the datasets is that each has gene expression levels separately constituting thousands of columns, and each consists of a quantitative clinical measurement considered as response variable. In addition, sample size of the datasets ranges from 15 to 98. Detailed information about datasets are shown in Table 1.

Some datasets had transformed counts. The log_2_ transformation was applied to nontransformed gene expression datasets. This normalization process adjusts the individual hybridization intensities to balance them appropriately to ensure that meaningful biological comparisons can be made [11]. In the next step, genotypes containing excessively many missing observations were excluded from the datasets.

Feature selection is another important step in selecting a small number of predictor variables having significant effects on the response variable, especially on high-dimensional gene data. Since the number of features is much larger than the sample size, classical methods such as ordinary least squares fail to fit the linear regression model. In addition, it is assumed that only a few of the features (genes) are actually associated with the response values. Hence, we must first identify the genes that are responsible for the dose of a clinical measurement to reduce the number of effective genes [12]. An iterative sure independence screening (SIS) feature selection method was conducted for this. When the model assumptions are not satisfied, SIS can miss important predictors. To overcome this problem, Fan and Lv [13] proposed iterative SIS to enhance methodological power. Iterative SIS could detect the combination effects of some marginally weak genes with the response variable by conducting SIS and lasso regression interactively. The basis is to apply large scale variable screening iteratively followed by moderate-scale careful variable selection [12, 13].

### 2.2. Regression Methods

First, we considered clinical-dose values (*y*) as response variable and gene expression profiles as predictors (*x*). Then, we identified small sets of predictor genes using iterative SIS method and built regression models ($\hat{y}=f(x,\beta )$), where $\hat{y}$ is the predicted dose, *x* is gene expression profiles, and *β* is a coefficient vector. These models were applied to predict clinical-dose values using only predictor gene expression data. For dose prediction, we used 6 regression models (SVR-linear, SVR-polynomial, SVR bagging, RT, RT bagging, and RT boosting). Training data (70% of data) was utilized to determine the optimal value of parameters and fit the models. Finally, model performance was estimated using the remaining (testing) data.

#### 2.2.1. Support Vector Regression (SVR)

Unlike the classical regression methods, SVR focuses on minimizing the generalization error instead of minimizing the observed training error. Vapnik has defined an *ε*-insensitive loss function (*ε*-SVR) to generalize support vector algorithms to apply to regression situations. The purpose of *ε*-SVR is to find the function *f*(*x*) with a maximum deviation of *ε* from the *y*_*i*_ target values for all training datasets. The linear function condition of *f*(*x*) is defined as follows:

(1) $$\begin{array}{c} f\left(\;x\;\right)=\langle \;w,x\;\rangle +b,\;w\in N,\;b\in R, \end{array}$$

where *w* is the flatness of the function and the following optimization problem is solved to minimize the ‖*w*‖^2^ Euclidean form.

Objective is

(2) Min: 12W2+C1N∑i=1NLεYi,fXi,

(3) LεYi,fXi=0Yi−fXi≤εYi−fXi−εotherwise.

Equation (3) is the experimental error measured with the *ε*-insensitive loss function [14]. As long as the errors are less than *ε*, they are ignored (considered zero). The *C* > 0 regularization parameter defines the balance between the flatness of *f* and the tolerated *ε* [14, 15]. Defining the kernel function, *K*(*X*_*i*_, *X*_*j*_) provides the objective function for the nonlinear solution:

(4) fx=∑i=1Nαi−αi∗Kxi,x+b,w=∑i=1Nαi−αi∗xi,

where *α*_*i*_ and *α*_*i*_^*∗*^ are Lagrange multipliers.

#### 2.2.2. Regression Trees (RTs)

The purpose of RT analysis is to explain a continuous response variable *Y* by explanatory variable vector *X* = *X*_1_, *X*_2_,…, *X*_*n*_, which can be a random mixture of quantitative, ordinal, and nominal variables [16]. A tree is developed by considering a root node containing primarily all of the observations. Observations in this node are sent to one of the two subnodes (left and right) using a split point on a single explanatory variable. The binary split process is applied repeatedly to its output until it reaches some stop criterion.

The response variable in each area for a split of *R*_1_, *R*_2_,…, *R*_*m*_ as the *m*th area is modelled as a constant *c*_*m*_ [17]:

(5) $$\begin{array}{c} f\left(\;x\;\right)=\overset{M}{\underset{m=1}{\sum }}c_{m}I\;\left(x\in R_{m}\right). \end{array}$$

Here, *I*() is the indicator function and *c*_*m*_ is the prediction value for *R*_*m*_. Using the minimization criterion of the sum of squared errors ∑(*y*_*i*_ − *f*(*x*_*i*_))^2^ reveals that the best value of ${\hat{c}}_{m}$ is the average of *y*_*i*_ values in the region *R*_*m*_:

(6) $$\begin{array}{c} {\hat{c}}_{m}=average\left(\;y_{i}\;|\;x_{i}\in R_{m}\;\right). \end{array}$$

In the RT formation step, two regions are created by applying a greedy algorithm for each explanatory variable *j* and split point *s*. Then, the first splitting variable *j* and split point *s* are obtained by solving the the following [17]:

(7) minj,s minc1⁡∑xi∈R1j,syi−c12+minc2⁡∑xi∈R2j,syi−c22.

#### 2.2.3. Ensemble Methods

Ensemble methods aim to improve the estimation performance of a given statistical learning or model-building technique. The basic principle of ensemble methods is to create a linear combination of model fitting methods instead of using only a single method. Bagging and boosting are two examples of aggregation methods used to increase the accuracy of classifiers or estimators. Yang et al. [18] proposed that ensemble methods such as bagging and boosting are effective in dealing with classification in high-throughput biological experiments. Therefore, such methods have been preferred recently owing to their special advantages in dealing with small sample size, high dimensionality, and complex data structures [8].

Classical bagging yields more robust and accurate models using bootstrap resamplings of the training data [18, 19]. The bagging procedure consists of two steps. First, bootstrap samples are drawn from the original data to form training sets, from which multiple models are obtained. Then, these models are combined to make predictions [19]. It has been shown [20] that bagging is especially suitable for unstable models such as tree-structured models.

Boosting was first introduced in 1990 by Freund and in 1995 by Schapire to improve classification [20]. As in bagging, the estimators that create the ensemble are obtained by resampling data and are then combined with the majority vote in the boosting method. Resampling a training set in bagging does not depend on the performance of the previous estimators. However, in boosting, the sampling probabilities of the samples that have the most different estimation values compared to the observed values for the regression predictors are adjusted to be higher as members of the training set for the second step. The estimations are combined using the weighted median by assigning greater weights to the predictors that are more reliably related to the predictions [21]. Boosting regression trees (BRTs) provide a method that aims to improve the performance of a single model by fitting multiple models and combining them for estimation. BRT uses two algorithms: RTs and boosting [20–23]. Hastie et al. (2001), who first established the connection between boosting and optimization, recommend the gradient-boosting machine (GBM) [17]. GBM is based on the AdaBoost algorithm, a derivative decrease algorithm on a loss function *L*(*y*_*i*_, *f*(*x*_*i*_)) [24, 25]. Boosting minimizes this loss function by modelling the residuals at every step.

### 2.3. Simulation Data

In order to ensure the effect of the sample size on the used ensemble methods, simulation data have been derived according to selected genes utilizing iterative SIS method. The gene expression-dose dataset having access number GSE2409 with a sample size of *n* = 69 has been used for this purpose. This dataset has been randomly selected to be an example. The number of the selected *q*^*∗*^ = 16 genes with iterative SIS method then has been reduced to 8 important genes by considering multicollinearity and the significance of the regression coefficients. The simulation data have been derived from the multivariate normal distribution by using the correlation matrix between gene expression measurements and dose values. In order to capture the true relation structure in the dataset, the zero mean vector and the real correlation matrix were used [26]. Data were obtained through the mvrnorm() function of MASS packages in the R software. The first column of the obtained data is the standardized scores of the dose values considered as the response variable. After model fitting step, these values were converted into the original dose scores using the mean and standard deviation of raw dose values as follows:

(8) zi=yi−y−σy,yi=zi×σy+y−,

where *z*_*i*_ is the standardized score, *y*_*i*_ is the raw dose score, and $\overset{-}{y}$ and *σ*_*y*_ are the mean and the standard deviation of the raw dose scores, respectively. These data generation steps were repeated 500 times.

### 2.4. Parameter Setting

Function types whose model performance was evaluated for SVR were linear, polynomial, radial basis, and sigmoid. However, only the solutions of linear function providing the smallest RMSE estimation were considered in the results section.

Generalization performance of SVR depends on setting some hyperparameters *C* (cost), *ε* (epsilon) and *γ* (Gamma, specific to kernel function) well [14, 27, 28]. Optimization of *C* and *ε* for linear function was achieved while the optimization of all the three parameters for other nonlinear kernel functions was achieved simultaneously using tune() function in *R*. The valid search interval of *C* (cost parameter) in tune.svm() command was determined between 10^−2^ and 10^2^. On the other hand, the search interval of *γ* (gamma parameter) was determined between 10^−3^ and 10^2^ (that is, 6 search points). Searches for 0.05, 0.10, and 0.20 values of *ε* in the related ranges of *C* and *γ* parameters were conducted; then, the combination of parameters which gives the smallest prediction error (MSE) for 10-fold cross-validation was identified. Similarly, minimum number of observations in a node, complexity parameter, and node number with subdivision (interaction level) parameters for RT were searched using cross-validation. Optimized parameter values were fixed for the method versions within the same cycle. 

### 2.5. Statistical Analysis

Statistical analyses were performed in R 3.1.2 programming language. Each dataset was divided into training (70%) and test (30%: evaluation) sets to avoid overfitting. When creating the regression models, the R packages e1071, rpart, ipred, and gbm were used for SVR, RTs, RT bagging, and GBM, respectively. R code was written to build a SVR-bagging model. The average performances of 100 training/test divisions and 50 bagging repetitions were considered in the analyses.

The prediction performances of related models were compared with the root mean squared error (RMSE), mean absolute deviation (MAD), and coefficient of determination (*R*^2^) measures, defined as follows:

(9) RMSE=∑i=1ny^i−yi2n,R2=SSRSST=1−SSESST,SSE=∑i=1nyi−y^i2,SST=∑i=1nyi−y−2,MAD=∑i=1ny^i−yin,

where *y*_*i*_ and ${\hat{y}}_{i}$ are the observed and predicted values of the response variable, *n* is the observation number, and SSR is the sum of squared regression, SSE is the sum of squared errors, and SST is the sum of squared of total variation of *y*. 

## 3. Results

### 3.1. Real Data Results

First, regression-based iterative SIS feature selection was applied to the previously prepared datasets substantially reducing the number of irrelevant genes from the raw datasets. After feature selection, only a small percentage of selected genes in all of the datasets remained. Detailed information and the number of selected important genes related to the datasets can be found in Table 1.

Thereafter, training-test performances with 100 random splits of single, bagging, and boosting models of the prediction methods were evaluated for the datasets. Prediction results of the SVR, RT, SVR bagging, RT bagging, and GBM learning techniques for each dataset are also shown as average *R*^2^ estimates in Table 2. Except for the GSE10748 dataset (*R*^2^ = 0.367), *R*^2^ values were generally found to be sufficient. Overall, the best prediction performance was obtained by SVR in nine of 11 datasets. In nine datasets, after SVR, the second best accuracy performance was provided by GBM. Average *R*^2^ values changed from 0.37 to 0.97.

For the GSE1938 dataset, the two genes that most affected the mannose concentration were CWP1 and NCA3. These two genotypes together best explained the mannose concentration, approximately 96%. In another yeast dataset with access number GSE8982, a total of nine important genes were determined to predict the concentrations of the alpha mating factor pheromone. Selected genes included DIG2, GYP8, YHR097C, ACM1, LCB3, PRM4, PRM3, FYV10, and UTP7, and all had the best explained variation, approximately 74%. In the analysis of the GSE12817 dataset, important genes such as Adm, Aldob, Pkm2, Crem, and nod3l were identified. Their explanation success of glucose concentration was found to be approximately 97%. Similarly, in the rat dataset with access number GSE2409, the selected 16 genes were RGD1309228, Ccdc21, Setd5, RGD1303130, Vps26a, Sept2, BF396256, Adprhl2, BE109616, Phf3, Tfb2m, Lyplal1, Arg1, Ndufs2, Tmem208, and Nsun2. Together, they had the best *R*^2^ values for anticancer drug dose, approximately 81%. When the two human datasets (GSE14954 and GSE9539) were considered, their *R*^2^ values were found to be sufficiently high. The best average estimates were 80.4% and 86.7%, respectively.

Twenty-two genotypes affecting copper concentrations were determined as MCL1, DDX28, MRPS26, RPUSD2, DDIT3, PELO, SLC30A1, C20orf111, SOCS3, SOX9, DNAJB4, NXF1, MAF1, MAFG, AMOTL2, ADM, ZFAND2A, ZFAND5, DUSP1, TBCC, HPS6, and INTS5.

**Figure 1:**
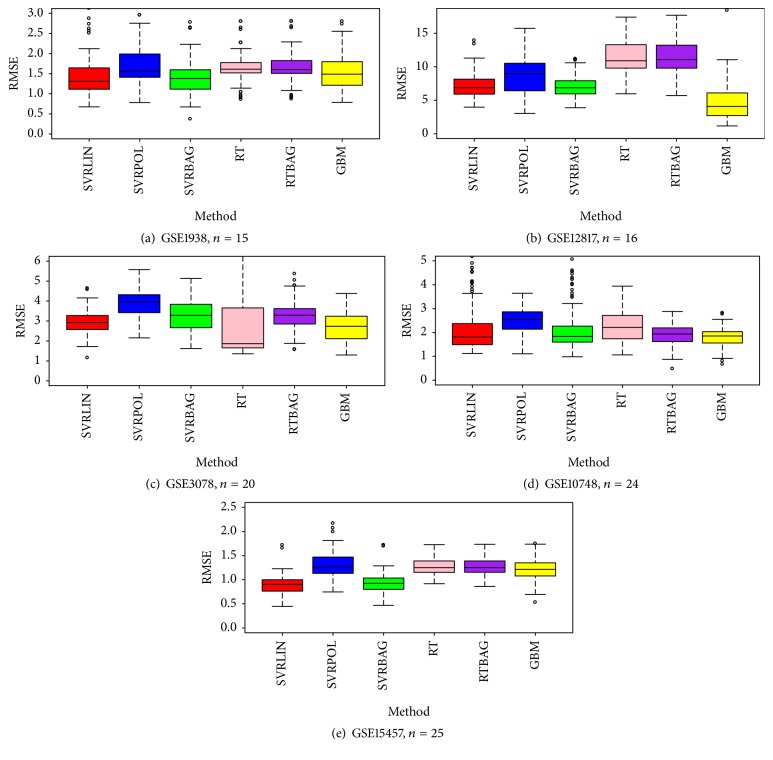
Box and whisker plots ((a)–(e)) of model RMSE performance for gene datasets with *n* ≤ 25 (100 repetitions): red box is SVR-linear solution, blue box is SVR-polynomial solution, green box is SVR-bagging solution, pink box is RT solution, purple box is RT-bagging solution, and yellow box represents GBM solution.

**Figure 2:**
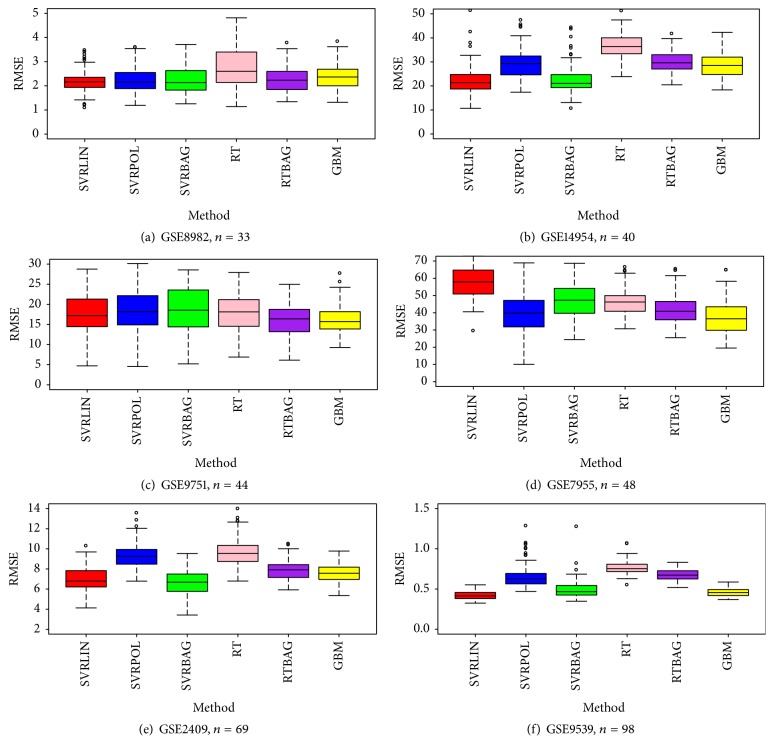
Box and whisker plots ((a)–(f)) of model RMSE performance for gene datasets with *n* > 25 (100 repetitions): red box is SVR-linear solution, blue box is SVR-polynomial solution, green box is SVR-bagging solution, pink box is RT solution, purple box is RT-bagging solution, and yellow box represents GBM solution.

The distribution ranges of the RMSE estimates generated with 100 repetitions for the method performances are presented in Figures 1 and 2 and categorized according to sample size. The plots obtained for five datasets in the *n* ≤ 25 condition and six datasets in the *n* > 25 condition are shown in Figures 1 and 2, respectively. It is obvious that the bagging RT performance had no advantage over RT consistently for all *n* ≤ 25 datasets (Table 2 and Figure 1). However, in datasets with *n* > 25 (Figure 2), bagging RT showed better performance compared to single RT in terms of RMSE and its standard error. On the contrary, this result is not valid for SVR performance. In both plots, SVR bagging did not outperform single performance for any dataset.

Within the SVR learning technique, a linear kernel function provided lower RMSE estimates than a polynomial one. Except for the dataset with ID number GSE7955, the polynomial model did not improve the average RMSE value (Figures 1 and 2).

### 3.2. Simulated Data Results

**Figure 3:**
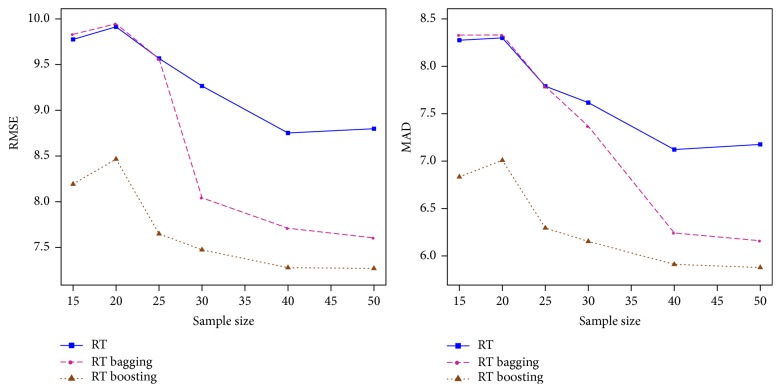
RMSE and MAD performances of single and ensemble of RT in 500 repetitions of the simulation data based on different training size. Blue straight line is single performance, pink dotted line is bagging performance, and brown dotted line is boosting performance of RT.

The selected 8 genes for GSE2409 dataset were obtained as Alg1, BF282239, AW920082, Ptpn12, BE103975, Prickle2, Nsf, and Phf3. In addition, the significance values of regression coefficients regarding them were found as *p* = (0.0018,0.0077,0.0160,0.0053,0.0122,0.0074,0.0687, 0.0694). Standard dose scores obtained based on (8) for selected database (GSE2409) were converted to raw dose estimations using dose mean (13.60) and its standard deviation (9.59). The results estimated by 500 repetitions of the simulation data are given visually in Figure 3. When these results are examined, it can be seen that the training set dimension of *n* = 25 is a cutoff point for the bagging method.

When the training set size is *n* ≤ 25, the bagging performance (pink dotted line) and single performance (blue straight line) of RT are very close; however, when *n* > 25, error predictions of bagging and boosting steadily decrease, but boosting gives a better performance compared to the others. These results are consistent with those of the original datasets.

## 4. Conclusions

In this study, the performances of two commonly used machine learning techniques, SVR, and RT, and their ensembles in predicting various dose values were compared on high- dimensional microarray datasets. A total of 11 real datasets obtained from the GEO database were used. Analysis of various dose values based on microarray gene expression data identified common genes found in our study and the referenced studies. For instance, the study that analysed rat pancreatic islets cultured for 18 h in 2, 5, 10, or 30 mM glucose concentrations of raw data with accession number GSE12817 identified the 40 genes most affected by glucose [29]. These included 16 upregulated genes, 19 downregulated genes,and five genes with a V-shaped profile. Aldob, Txnip, Crem, Adm, and Fos were found among the 16 most upregulated genes known to be strongly induced by high glucose. Similarly, five genotypes were determined in our analysis as having the greatest effect on glucose concentration. These were the Adm, Aldob, Pkm2, Crem, and nod3l genotypes. Moreover, the explanation success of glucose concentration by these five genotypes was found to be approximately 97%. The GSE14954 dataset belonged to 20 male and 20 female participants with ages between 45 and 60 years and body mass indices ≥25 kg/m^2^. All participants received two different diets and then their LDL-cholesterol concentrations and insulin sensitivity levels were compared [30]. It was found that a saturated fatty acid diet resulted in changed expression of 1523 genes, whereas a monounsaturated fatty acid diet resulted in changed expression of 592 genes. In our study, we demonstrated approximately 80% prediction success of LDL-cholesterol concentrations using only genetic information of all the participants. The genotypes most affecting LDL-cholesterol concentrations were identified as LOC100289611, PKDCC, DCTD, LASS6, KDSR, UBXN6, PDE8B, DMWD, SPATA20, and RGS7BP. For the GSE9539 dataset, human HepG2 cells were treated with 100, 200, 400, or 600 *μ*M copper sulfate. A related study [31] identified a total of 2257 differentially expressed genes by fold change. Of these, the upregulated genes were found to be HSPs, BAG3, SOCS3, GADD45G, GCLM, VLDLR, CYR61, DUSP1, DUSP5, FOS, EGR1, MAFB, NR4A1, PROP1, TGFB1, DNAJB1, ADM, and DDIT3. On the other hand, MCL1, IFRD1, JAG1, MAFB, CAP1, and FGD6 genotypes were among the downregulated genes. In our analysis, 22 genotypes affecting the copper concentrations were determined. Seven of these, MCL1, DDIT3, SOCS3, DNAJB4, MAFG, ADM, and DUSP1, were found to be similar to those reported by Song et al. [31]. An important finding related to this dataset was that it had considerable predictive success (*R*^2^ = 0.867). Harrill et al. [32] used two penalized regression methods to detect dose-dependent changes in the gene transcription of rat frontal cortex. For deltamethrin and permethrin doses, 95 of 109 (87.1%) and 53 of 89 (59.5%) probe sets passed the ANOVA significance threshold. Egr1, c-fos, Gpdl, Fkbp51, Hsp27, Camklg, Bdnf, Rassf5, and LOC689415 were found among the genes upregulated by deltamethrin and permethrin. On the other hand, Ddc, Slc39a8, Pldl, LOC682926, Slit2, Klf4, and Bves were found among the genes downregulated by deltamethrin and permethrin. In this study, we identified 14 genes that can most affect dose values. Among them, c-fos, LOC689415, and Bdnf were found to be similar to those reported by Harrill et al. [32]. However, the deltamethrin dose *R*^2^ success of these 14 genotypes was about 55%.

When the prediction performances were compared, the fact that SVR performs better than other methods closely resembles the results of some other studies [6, 14, 33]. While the lowest dose *R*^2^ was obtained as 0.367, the highest *R*^2^ was 0.978.

An important finding of this study is that the dataset size of *n* = 25 was identified as a cutoff point for RT bagging. When RMSE estimates from RT to RT bagging were examined with changing sample size, it was seen that bagging did not improve single RT performance up to the datasets with sample size of 25. However, the *R*^2^ estimates of RT bagging gained consistency from the dataset with ID code GSE8982 (*n* = 33), and this improvement became more visible with an increase in the number of observations. Consequently, GBM provided more optimistic performance in datasets with *n* ≤ 25 compared to RT bagging. It was also found that the results obtained from the simulated data were consistent with the application of real gene data. However, this condition is not valid for SVR performance because SVR bagging did not outperform single SVR. When SVR performance was taken into consideration, in either condition (*n* ≤ 25 or *n* > 25), bagging could not provide a significant reduction in the RMSE average or variance. This finding is consistent with the results of some other studies in the literature [22, 34].

Furthermore, frequent utilization of iterative SIS feature selection for genomic data was supported in this study, because it enables the selection of a smaller number of genes to contribute to regression estimation from thousands of raw genes.

## Figures and Tables

**Table 1:** Summary information and number of selected important genes of the real datasets.

Data/ID	Organism type	Response variable	Number of observations	Total genes	Selected genes
GSE1938	Yeast	Mannose concentration	15	8714	2
GSE12817	Rat	Glucose concentration	16	24606	5
GSE3078	Rat	Hydrogen peroxide	20	14102	6
GSE10748	Rat	D-serine dose	24	24606	7
GSE15457	Rat	Hypochlorous acid	25	27276	4
GSE8982	Yeast	Alpha mating factor pheromone	33	6191	9
GSE14954	Human	LDL-cholesterol concentrations	40	16401	10
GSE9751	Rat	Chlorpyrifos (CPF) dose	44	13429	11
GSE7955	Rat	Deltamethrin dose	48	24606	14
GSE2409	Rat	Anticancer drug dose	69	7987	16
GSE9539	Human	Copper sulfate	98	18211	21

**Table 2:** Average *R*^2^ estimates of learning techniques with 100 repetitions.

Dataset	SVR (lin)	SVR (pol)	SVR bagging	RT	RT bagging	GBM
GSE1938	0.962^*∗∗*^	0.874^*∗∗*^	0.941^*∗∗*^↓	0.838^*∗∗*^	0.724^*∗∗*^↓	0.943^*∗∗*^↑
GSE12817	0.978^*∗∗*^	0.925^*∗∗*^	0.963^*∗∗*^↓	0.792^*∗∗*^	0.847^*∗∗*^↑	0.945^*∗∗*^↑
GSE3078	0.639^*∗*^	0.583^*∗*^	0.538^*∗*^↓	0.759^*∗∗*^	0.739^*∗∗*^↓	0.760^*∗∗*^↑
GSE10748	**0.367**	0.289	0.303↓	0.254	0.290↑	0.263↑
GSE15457	0.879^*∗∗*^	0.407^*∗*^	0.769^*∗∗*^↓	0.720^*∗∗*^	0.231↓	0.884^*∗∗*^↑
GSE8982	0.740^*∗∗*^	0.686^*∗∗*^	0.574^*∗∗*^↓	0.593^*∗∗*^	0.616^*∗∗*^↑	0.720^*∗∗*^↑
GSE14954	0.804^*∗∗*^	0.653^*∗∗*^	0.710^*∗∗*^↓	0.426^*∗*^	0.584^*∗∗*^↑	0.781^*∗∗*^↑
GSE9751	0.552^*∗∗*^	0.363^*∗*^	0.457^*∗*^↓	0.135	0.275↑	0.275↑
GSE7955	0.549^*∗∗*^	0.466^*∗∗*^	0.443^*∗*^↓	0.198	0.300^*∗*^↑	0.325^*∗*^↑
GSE2409	0.814^*∗∗*^	0.263^*∗*^	0.714^*∗∗*^↓	0.440^*∗∗*^	0.538^*∗∗*^↑	0.644^*∗∗*^↑
GSE9539	0.867^*∗∗*^	0.700^*∗∗*^	0.823^*∗∗*^↓	0.552^*∗∗*^	0.664^*∗∗*^↑	0.833^*∗∗*^↑

Bold indicates the best performance; ↑: increase in *R*^2^; ↓: decrease in *R*^2^ compared to single performance. *∗∗* and *∗*: significant at 0.001 and 0.05 level, respectively. SVR (lin), SVR (pol), and RT are single performances, while the others are ensemble performances of the methods.
